# ‘Single‐handed care’ initiatives and reviews of double‐handed homecare packages: A survey of practices in English local authorities with adult social care responsibilities

**DOI:** 10.1111/hsc.13980

**Published:** 2022-09-01

**Authors:** Phillip J. Whitehead, Leigh Rooney, Jane Adams‐Thomas, Catherine Bailey, Marie Greenup, Carole Southall, Anne Raffle, Tim Rapley, Stephanie Whittington

**Affiliations:** ^1^ Population Health Sciences Institute Newcastle University Newcastle upon Tyne UK; ^2^ Occupational Therapy Team Nottingham City Council Nottingham UK; ^3^ Department of Nursing, Midwifery and Health Northumbria University Newcastle upon Tyne UK; ^4^ Occupational Therapy Team Sunderland City Council Sunderland UK; ^5^ Department of Social Work, Education and Community Wellbeing Northumbria University Newcastle upon Tyne UK; ^6^ Elders Council Newcastle upon Tyne UK

**Keywords:** double‐handed care, homecare, moving and handling, single‐handed care, social care

## Abstract

International health and social care systems are experiencing unprecedented pressure and demand. ‘Single‐handed care’ initiatives seek to identify whether all or part of a homecare package involving more than one care worker can be safely reduced to a single worker. Little is known about these initiatives across local authorities. The aim of this study was to identify, describe and explain current processes and practices for single‐handed care initiatives and double‐handed homecare reviews. An electronic survey link was sent to each local authority with social care responsibilities in England. The questions covered a range of areas in relation to single‐handed care processes and included a combination of pre‐coded and free‐text responses. Responses were received from 76 (50%) local authorities. Findings were that over 12,000 reviews were reported within a year with a median of 141 (IQR 45–280) from 53 authorities that provided figures. Reviews were usually led by a local authority occupational therapist. On average, 540 min was spent per review, including conducting and organising the review, documentation, and travel. In nearly half the authorities, double handed care remained at least partially in place following at least 80% of the reviews and remained wholly in place following at least 60%. Local authorities also reported some resistance from homecare providers when implementing single‐handed care. The findings have confirmed anecdotal evidence that reviews of double‐handed homecare packages are common practice within local authorities. Given the amount of time taken with these reviews, and paucity of evidence on outcomes for people receiving them, further research should evaluate this.

**What is known about this topic**

- Single‐handed care initiatives/double‐handed reviews seek to reduce all or part of a care package involving more than one care worker to a single care worker.
- They are reported to lead to increases in wellbeing and dignity for people using services and to cost savings.

**What this paper adds**

- Fifty‐three local authorities in England carried out over 12,000 reviews of double handed care packages in one calendar year, and all planned to maintain or increase the amount completed.
- Reviews are usually led by a local authority occupational therapist and each, on average, is more than a day's work.
- In nearly half the authorities, double handed care remained wholly or partially in place following at least 80% of the reviews and remained wholly in place following at least 60%.

## 1 INTRODUCTION

International health and social care systems are under sustained pressure. Challenges include exponential growth in demand, an ageing population, and real‐term budget cuts since the global financial crisis of 2007 (Robertson et al., 2014). In England, health and social care systems were experiencing pressures before the COVID‐19 pandemic (National Audit Office, 2021; Warner & Zaranko, 2021) which have since been exacerbated. Enabling people with health and social care needs to remain safely supported at home is a key priority, although a common barrier to timely discharge from acute care is a lack of available support within the community (Allan et al., 2021). In the UK, The Care Act 2014 placed a statutory responsibility on local authorities with social care responsibilities in England to provide services which prevent or delay the need for care and support, or minimise the need for additional care and support (Department of Health, 2014). One such initiative that is reported to have become commonplace amongst local authorities during this period is ‘single‐handed care’.

The terms ‘single‐handed care initiative’ and ‘double‐handed homecare reviews’ are used throughout this article. A ‘single‐handed care initiative’ often involves a specific focussed project within a local authority which seeks to reduce all or part of a care package involving multiple care workers (Harrison, 2017), commonly from two to one worker (Buckinghamshire Council, 2021). Care packages involving two workers – ‘double‐handed care’ – may be indicated when particular pieces of moving and handling equipment are used, such as a mobile hoist, or when the moving and handling procedures are particularly complicated. Double‐handed homecare packages became increasingly common as people with higher dependency needs, who may previously have been cared for in a nursing or residential home, were enabled and supported to remain safely in their own home with two care workers on each homecare call. However, although double‐handed homecare packages were once considered ‘solutions’ to social care ‘problems’, they have more recently become targets for intervention in their own right. ‘Double‐handed homecare reviews’ are usually conducted with the aim of determining whether the double‐handed homecare is needed on an ongoing basis, but may be conducted more routinely (i.e. not as part of a specific focussed project). Reasons for the growing prevalence of single‐handed care initiatives and double‐handed homecare reviews include cost savings (Charlton et al., 2015), rationalising the homecare workforce and advancements in moving and handling equipment (‘assistive technologies’) and associated techniques (Personal Care Consultants, n.d.). Reducing a care package from one to two workers is also purported to increase dignity for the person using the service (Buckinghamshire Council, 2021). However, the exact mechanism by which this might increase dignity is often unspecified, although it is often linked to better relationships with the care workers and greater privacy (Buckinghamshire Council, 2021; Royal College of Occupational Therapists, 2019).

The sustainability of the English social care system is one of the top national priorities for national and local governments. Age UK (2019) reported that the number of older people with some level of unmet social care needs is 1.5 million, the equivalent of one in seven of the older population. Recruitment and retention of the social care workforce is particularly challenging with the care worker vacancy rate about 12% (Skills for Care, 2021); nearly one in three social care staff in England left their jobs in 2017/18 (The Kings Fund, 2018). Pre‐pandemic projections suggest that 320,000 additional social care staff will be required in England by 2029/30 (The Kings Fund, 2018). One potential ameliorator to the supply problem with the homecare workforce is through single‐handed care: enabling people to be safely cared for by one person in a situation which may previously have required two.

A review, undertaken by Phillips et al. (2014), critically examined the perceived need for two homecare workers in the context of legislation. It synthesised case studies and reported ‘conversions’ or reductions between 25% and 44% from double to single‐handed homecare across the three local authority case study sites. However, the review also concluded that outcomes for service users and their families, including qualitative experiences, were not available. Harrison (2018) reported a survey, largely of occupational therapists and moving and handling professionals, which identified some misunderstandings and barriers to the implementation of single‐handed care which included concerns about processes and lack of confidence. This report also pooled figures from ten local authorities which reported reductions in care in 17%–44% of cases.

From a theoretical perspective, the trajectory of disability might be modified by the provision of either assistive technologies, personal assistance from a carer or care worker, or both (Hoenig et al., 2003). The use of assistive technology has grown exponentially within health and social care and there is a widespread literature examining the effectiveness of various technologies across a range of health and social care outcomes (Brims & Oliver, 2019; National Institute for Health Research, 2018). Literature also suggests that use of assistive technology may be associated with fewer hours of personal assistance (Hoenig et al., 2003) or informal care hours (Agree et al., 2005). We were unable to find any empirical studies which have specifically examined the views of people who had experienced reductions from two to one carer workers through single‐handed initiatives.

Although there is a dearth of academic literature, anecdotally it is clear that single‐handed initiatives are common across local authorities. We carried out a search for ‘single‐handed care’ ‘single‐handed care’, ‘single‐handed homecare’, ‘single‐handed homecare’ and ‘moving with dignity’ in Google for domains ending in “gov.uk”. Accounting for duplicates, this revealed 211 documents from 50 local authorities in England. However, there is a lack of information on processes and practices within and between local authorities and many initiatives may be pilot schemes or time limited and the ongoing prevalence of the practice across local authorities is not known. The aim of this study was therefore to identify, describe and explain current processes and practices for single‐handed care and double‐handed homecare reviews across local authorities with adult social care responsibilities in England. In doing so, the intention was to synthesise key areas of similarity and difference and establish a definition of ‘usual care’ in which to benchmark future comparative evaluations.

## 2 METHOD

We developed a bespoke questionnaire for this study as no existing instrument was available. This involved discussion amongst all authors, which included researchers, social care practitioners and a lay contributor. We also developed a working group comprised of practitioners with experience (occupational therapists, social workers and homecare workers) and a family member with lived experience. The working group commented on the development of the instrument and the interpretation of the findings. The questionnaire was divided into sections which covered the following areas: number of reviews completed, identification of eligible reviews, time‐point for reviews and time spent completing them, review procedures, review outcomes, involvement of people using services and their families and plans for future reviews. Favourable ethical opinion was provided by the Health Research Authority via the West Midlands – Coventry and Warwickshire Research Ethics Committee (Ref: 19/WM/0224). The survey also received endorsement from the Association of Directors of Adult Social Services (ADASS).

A draft version of the questionnaire was initially piloted with members of the research team, the working group and then local authority occupational therapists from collaborating organisations and through our existing contacts. Minor changes were made to the design of the survey following piloting. Before sending out the questionnaire, we made telephone contact with each of the 151 local authorities with social care responsibilities in England in order to attempt to identify a named contact to whom it would be appropriate to send the survey. We were able to identify named contacts for 148 local authorities and a generic contact address for the remaining three authorities. An electronic link to the questionnaire was emailed in June 2020 with a covering letter and instructions on how to complete it. A copy of the questionnaire can be obtained from the corresponding author. The survey was completed online at Online Surveys (onlinesurveys.ac.uk), with each local authority having a unique access login that enabled the survey to be completed only once per authority. Each login contained a unique username identifier specific to a particular local authority, and this link was recorded in a spreadsheet. This allowed a record to be kept of which local authorities had completed the survey, and a reminder email was sent 2 weeks later to those who had not completed. In order to maximise the response rate, subsequent emails advertising the survey were sent through the Royal College of Occupational Therapists' Principal Occupational Therapists in Social Care list and Association of Directors of Adult Social Services asking local authorities to make contact with the researchers if they were interested in participating. After the survey closed, and before the data within the survey was examined, the link between the unique identifier and the name of each local authority was broken in order to anonymise the data.

Analysis of the questions which were pre‐coded into categorical responses was completed within Online Surveys. Analysis of questions which yielded continuous numerical data were analysed within Microsoft Excel. Data from the categorical and numerical responses are presented using descriptive statistics in the text and supplemented with charts and tables. Several questions gathered free text responses, including those where an “other” option was given. For some questions, the data were simple enough to derive an answer reading and noting the responses. However, where the data were more complex, a thematic analysis was carried out, which involved coding data into units according to well‐established qualitative analytical principles of categorisation and comparison (Braun & Clarke, 2012; Merriam & Tisdell, 2015). Seven questions were analysed in this way with the aid of NVivo (version 12) computer software.

## 3 FINDINGS

Seventy‐six questionnaires were returned, slightly above a 50% response rate. Fifty‐five (72%) responses were from single tier authorities with 21 (28%) from the upper tier of two‐tier authorities. Seventy (92%) local authorities reported that they completed reviews of double‐handed homecare packages in the year 2019. Thirty‐one (44%) local authorities carried out reviews as part of a single‐handed care project or initiative, 22 (31%) were combined with other review processes and 13 (19%) were completed as standalone reviews^1^; thirty‐nine (56%) carried out reviews on both a standalone basis and combined with other review processes (i.e. they had both single‐handed care initiatives and also combined them with other review processes).^2^ Thirty‐nine (56%) reported that double‐handed reviews were carried out on a routine basis, 18 (26%) on an ad hoc basis, and 13 (18%) ‘other’. Those providing additional details for ‘other’ reported that reviews were completed both routinely and ad hoc, when time allows, at the request of a social worker or care manager, or when the need for double‐handed care was identified. Some also provided additional comments on the incorporation of single‐handed care into routine practice and some of the difficulties in identifying those appropriate for timely reviews.

For those authorities (*n* = 31) which reported carrying out reviews as part of a single‐handed project or initiative, we asked them to provide free text responses for further information. Five areas were identified. First, projects were discussed in terms of being an initial *exploration* of single‐handed care practice/double‐handed care reviewing, sometimes taking the form of pilot projects. If successful they were then adopted as “business as usual”. Second, projects were framed in terms of an aim to *reduce and avoid* double‐handed care. However, the language was often couched in terms of determining the “appropriate” level of need. Third, reflecting the emphasis on reduction and avoidance, respondents noted that reviews were rarely triggered automatically in response to a *reduction* in double‐handed care. Only one response noted that, in addition to increases to double‐handed care triggering a review becoming standard practice, they have recently seen more “referrals to OT [occupational therapy] to review requests for reductions too”. Fourth, *justification* for the project was given encompassing three areas: the potential to save money, improvements in quality of life of life for people using the service, and to increase capacity within the homecare system. The fifth area concerned issues of implementation. This stressed the importance of using equipment and moving and handling techniques to achieve single‐handed care, and of the need to train staff in this regard. Respondents also stressed the importance of all stakeholders working together. Challenges were reported with homecare providers being highlighted as sometimes initiating “resistance” to single‐handed care, possibly with a “risk averse” approach rooted in “blanket policies” and “myths” related to moving and handling policies and procedures.

**FIGURE 1:**
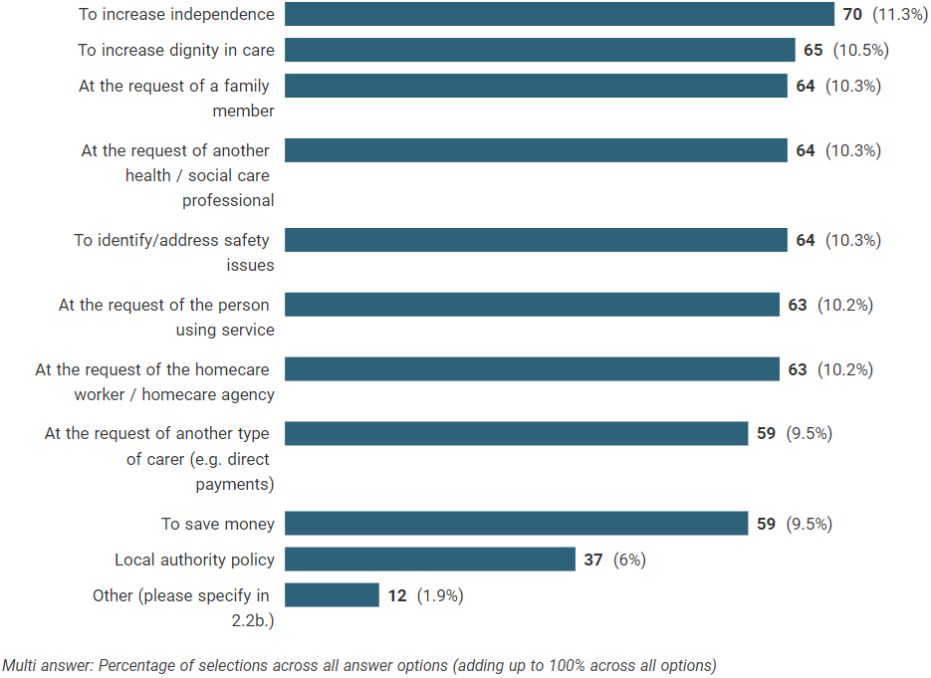
Reasons for completing reviews

Fifty‐three authorities provided figures for the number of reviews of double‐handed homecare (either standalone or combined with other processes) completed in 2019 (19 provided actual figures, 34 provided estimates and 17 did not know the figure). A total of 12,129 reviews were reported with a range of 12 to 2000, median 141 (IQR 45–280). Sixty‐nine percent of all authorities reported that this review year was typical. We asked why double‐handed homecare reviews were completed within the authority, and Figure 1 shows the responses for all reasons.^1^ All authorities stated they completed reviews in order to increase independence, with the next most common reasons being to increase dignity in care, at the request of a family member, at the request of another professional and to identify or address safety issues. Having asked respondents to select all reasons for which they might complete a review, we then asked respondents to indicate the most common reason. Interestingly, the most common reason overall was because it was local authority policy, but only 53% stated that they had a local authority policy to complete reviews. This indicates that where it is a local authority policy reviews are likely to be completed. The next most common reasons were at the request of the homecare agency and to increase independence.

**FIGURE 2:**
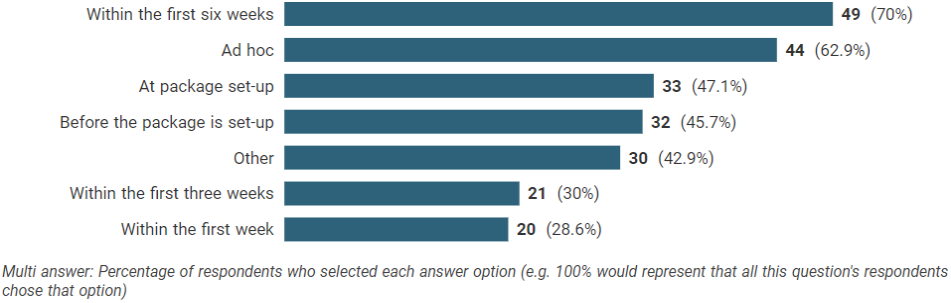
Timepoint for completion of reviews. Responses to ‘Other’ included 11 which mentioned ‘annual review’, 5 which specified ‘according to need or the situation’, and one each for ‘as part of a manual handling assessment’, ‘no specific timescale’, ‘after the introduction of equipment’, ‘when capacity allows’, ‘following discharge to assess’, ‘6 monthly’, ‘in response to safeguarding‘, due to a specific project’, ‘due to an increase in care’, ‘following a short term admission’. Four responses were not clearly attributable to the question.

Figure 2 shows the timepoint in the person's episode of care at which the reviews were completed. This was a multipoint answer meaning that respondents could select all options that applied and there appears to be a great deal of variation within and between the local authorities in the time at which reviews are completed. Reviews were most commonly reported to be completed ‘within the first six weeks’ by 49 (70%) of respondents, followed by ‘ad hoc’ by 44 (63%) and ‘at package set up’ by 33 (47%). Table 1 shows the time spent, on average, at each stage of the review process. An average of three visits to the person's home were completed per review; although there was a range of 1–13. There was an average of 60 min per visit, with averages of 60 min organisation time, 180 min completing the documentation and 40 min travelling. This gives an approximate average total time of 540 min (3 × 60 min visits each with 40 min travel plus 60 min organisation and 180 min documentation). This is the equivalent of more than a full day's work; however, this figure should be interpreted cautiously as it is the aggregate of the averages. This figure will also be higher where there are multiple staff members involved. Table 1 also shows that there is some wide variability between the time spent on each aspect of the review process, particularly organising and documenting the review, indicating that procedures and practices may vary considerably between authorities.

**TABLE 1:** Time spent on double‐handed homecare reviews.

	Median	IQR	Range
Number of visits per review	3	2–4	1–13
Time spent organising (minutes)	60	30–120	12–1800
Time per visit per staff member in the person's home (minutes)	60	60–81	30–180
Time completing documentation (minutes)	180	105–260	12–1800
Travel time per visit	40	25–60	10–120

**FIGURE 3:**
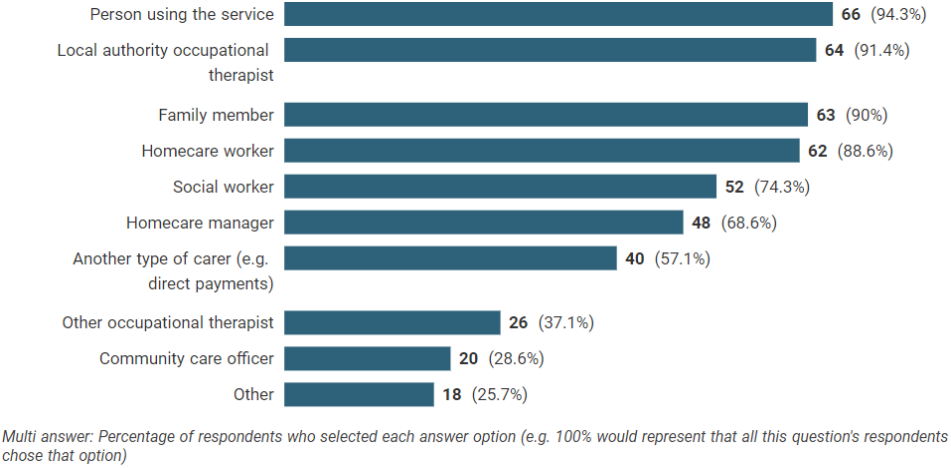
People involved in the review in routine practice. Other options included 4 x ‘manual handling or back care advisor’, 3 x ‘equipment store or equipment provider’, 3 x ‘district nurses’, 3 x ‘physiotherapist’, 3 x ‘day centre staff / manager’, 2 x ‘equipment rep’, 2 x ‘advocate’, 2 x ‘specialist nurses e.g. intermediate care or tissue viability’, and 1 each of ‘brokerage ‐ for commissioning’, ‘OT for specific project’, ‘lasting power of attorney’, ‘friends’, ‘people from statutory services’, ‘GP’, ‘housing’, ‘Reablement care assistant’, ‘senior manager (for funding approval)’. Note that some respondents provided more than one category of person for this option.

Figure 3 shows the responses to the question *“In routine practice, which of the following people would be involved in a double‐handed homecare review?”* The person using the service (94%), local authority occupational therapist (91%) and family member (90%) were the most common. It is noteworthy that four (6%) local authorities who answered this question did not report that the person using the service was involved in the review. Local authority occupational therapists (87%) were most commonly reported to lead the review and assume overall responsibility, followed by social workers (45%).^3^ Fifty‐three percent of authorities reported that a subsequent follow‐up review or check visit was “always” arranged, followed by 24% reporting “often.” Ten percent reported only completed follow‐up visits if the care package had been reduced.

**FIGURE 4:**
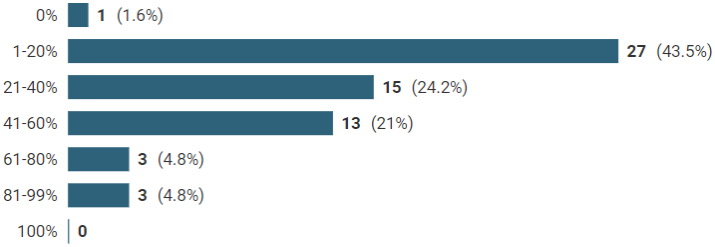
Homecare packages wholly or partially reduced to single‐handed care following a double‐handed homecare review.

Figure 4 shows homecare packages which were wholly or partially reduced to single‐handed care following the review (62 local authorities answered this question, 27% provided actual figures and the remainder were estimates). A partial change means the care package involved one worker sometimes but not every time. The most common response was ‘between 1% and 20% of homecare packages being wholly or partially reduced’ reported by 44% of local authorities. In addition, we also asked what proportion of homecare packages remained *wholly* double‐handed care following the review process. Fifty‐eight answered this question and 26 (44%) local authorities reported that this was greater than 60%.

Respondents were asked to provide a free text answer to the question “how are review outcomes agreed?” It was reported that this was through interaction between stakeholders: people using the service, family members, occupational therapists, social workers, and homecare staff. Interactions included “discussion”, “conversation”, “negotiation”, “collaboration”, and “consultation” amongst at least two stakeholders. Responses noted a “partnership” amongst stakeholders in this process, which is undertaken “jointly”. For staff stakeholders this might be seen to require respect for their “professional autonomy”, and for a “person centred approach” to be taken with service users. Consistent with the responses to the pre‐coded questions, occupational therapists were reported to play a key role in leading the process.

We asked respondents to provide free text response to “what happens if there is a difference of opinion amongst those present?” Seven overlapping areas were identified. First, there is usually some further interaction about the differences to reach agreement. This may take the form of further interaction between stakeholders, with responses noting that “we would discuss further” and have “[m]ore joint visits/meetings/case conference[s]”. The second area concerns the importance of *evidence* in formulating a decision. A key source of evidence is a moving and handling risk assessment, which one response noted “will have the last say” in the decision, suggesting that this is invoked to support any difference of opinion. The third area concerns the involvement of additional persons. These additional persons might act to provide further evidence, such as another occupational therapist performing a second moving and handling risk assessment. Involvement of more senior staff was also reported.

The fourth area indicates the use of a trial period to help address differences of opinion. This allows stakeholders “to test out” proposed changes to the care package. Thus, one response noted that the trial period “allows the individual or family member to test out their reduced care package for a few days to highlight their own strengths and restore confidence.” The use of the trial period evidence as a means of orientating (by means of personal experience) to single‐handed practices can be seen as a part of the fifth area: providing opportunity for adjustment to single‐handed practices. In particular, the building of “confidence” in these practices is noted.

As discussed in the analysis of data from the other questions, homecare staff are identified as a particular source of resistance to single‐handed care practice. Disagreement between those proposing single‐handed care and homecare providers might be managed through some sort of formal pressure applied to homecare agencies to comply with single‐handed care proposals, and this constitutes the sixth area. This might involve severing links between the local authority and that homecare provider and the seeking of another provider. One response noted that homecare providers “work under the policies of [the local authority…and] this enable us to challenge the care providers[’] blanket rule that people need double‐handed [care] for certain pieces of equipment.” However, another response noted that taking a “hard line” with care providers might be difficult as “our dom[iciliary] care market is v[ery] fragile”, suggesting that simply replacing (or threatening to replace) the homecare provider is not necessarily a viable option. The final area involves a service user/their family funding additional care where they disagree with single‐handed care proposals. Some responses framed this in terms of service users/their family being given the *choice* to self‐fund (“able to choose”), but one response noted the “[e]thical dilemma” of whether “we should ask the person to contribute to the extra costs incurred by their preferences”.

All local authorities responding to a question about involvement of people using services and their families (70 responses) reported that these groups were involved in the double‐handed homecare review process. Sixty‐seven provided responses to the question “please tell us how [they are involved]” and three methods of involvement were identified. First, involvement of service users and their families was framed using a rhetoric emphasising their *centrality* in the review process. Here service users and families “are always at the centre of what we do”, “held central to the process”, “[f]undamental to all we do”, “and are “at the heart of the review”. This centrality was further emphasised by the assertion that service users and their families are “involved from the beginning of the process to the end” and “at all levels of the review”. This is enacted as an established method of “good practice” identified as a “person centred” or “customer centric” approach.

Second, involvement of service users and their families in the review process occurs through some sort of *dialogue* between them and other stakeholders, of “conversation” and “discussion” and indeed one response went further and suggested the primacy of the service user as an agent in the interaction (“[w]e would ask the person to direct as much of the assessment as possible”). However, this dialogue is often reported as local authority staff initiating conversation with service users and their families who are discussed in the passive voice. Thus, responses note that “[t]he therapists liaise with family” and that the “views of [the] client and their families is sought”, “obtained”, and “captured” by local authority staff. Reflecting this active/passive distinction, the third method of involvement concerns the flow of information *from* the local authority *to* service users and their families. Thus, the latter are kept “informed” by the former about the review processes. This might involve local authority staff “explaining to them” about these processes including “helping them to understand the benefits of single‐handed care”.

Thirty‐eight (51%) authorities reported that they planned to increase the number of double‐handed homecare reviews, twenty‐eight (38%) reported that they planned to maintain current level, and eight (11%) responded ‘other’ and provided comments that included indications that they were exploring options or were unsure of future plans. No authorities indicated that they planned to reduce the number of double‐handed homecare reviews.

## 4 DISCUSSION

Over half of all local authorities with adult social care responsibilities in England responded to this survey. Key findings suggest that reviews of double‐handed homecare packages are a common practice with over 12,000 completed within the 53 local authorities who provided data, with a median of 141. The majority of local authorities planned to increase these reviews, and none planned to decrease. Forty‐four percent of authorities had a specific project or initiative directed at this area of practice. One aim of this research was to establish a benchmark of ‘usual’ care across local authorities. Although our findings reveal widespread variation in many areas, it has been possible to synthesise some areas which might comprise usual care. The key overall similarity was that the reviews were usually led by a local authority occupational therapist in 87% of local authorities. Reviews consisted of an average of three visits to the person's home with an average time taken of 60 minutes per staff member per visit; the total average time was 540 minutes, including conducting the review, preparation, documentation and travel. There was particularly large amount of time spent on documentation at 180 minutes, with the greatest variability in this area. In over two‐thirds of local authorities, reviews were completed within the first six‐weeks of the person's episode of care. This finding demonstrates that the majority of local authorities complete reviews of double handed homecare packages early in the person's episode of care in order to review the need for two care workers on an ongoing basis.

All local authorities reported that the person using the service and/or their family were involved in the review process with references to working in partnership with the person; however, a small number of authorities reported that the person using the service was not involved. Many authorities reported that the person was at the heart of the process and central to it. This finding is consistent with wider policy – principally the tenets of The Care Act 2014 and personalisation agenda – and should be considered good practice within health and social care. Contributing a personalised approach to care is one of the key contributions that occupational therapists make to social care in the UK, according to the Royal College of Occupational Therapists (2019). Nevertheless, when synthesising responses across the questions included in the survey, there were some difficulties that were evident in this. For example, in the event of a difference of opinion between the local authority and the person, the person would be given the option of paying for their own preferred care arrangement. This is perhaps at odds with a person‐centred approach and would likely not be an option for some people using the service. It was also reported, in one response, that the risk assessment “will have the last say” in the decision, which might also be at odds with the person being at the centre.

Responses to how differences of opinion were resolved revealed some further potential tensions in the process between local authority reviewing staff and homecare agencies or providers; there were some references to “resistance” on the part of some homecare providers, which is consistent with previous reports (Harrison, 2017). These responses also alluded to the potentially fragile nature of the homecare market with suggestions that the local authority may review the providers' contract in the event of a difference of opinion. However, another response indicated that the fragility of which implied that this option was potentially not available to local authorities. Although single‐handed care initiatives are widely purported to reduce the pressure on the social care workforce these findings suggest this may not be straightforwardly so. Furthermore, this reflects the wider problems of supply and demand and the fragmented nature of the adult social care system in England (Eynon & Conroy, 2017). Although some tensions with homecare providers were alluded to, our findings also reveal that the second most common reason for completing reviews was ‘at the request of the homecare agency’. Thus, these findings might suggest that homecare agencies are becoming more ‘on‐board’ with singled handed care processes and are likely identifying and referring people who might be appropriate for them. It is also possible that this is changing over time as more homecare providers become familiar with the provision of single‐handed care and the use of specific techniques, such as assisting the person to roll single‐handedly or in the use of the specialist moving and handling equipment.

There is a lack of research on the outcomes of double‐handed care reviews with which to compare our findings. However, previous reports which included data from three (Phillips et al., 2014) and ten (Harrison, 2018) local authorities reported that reviews led to reductions in care packages in 17%–44% of cases. Our findings were consistent with this with 90% of authorities reporting reductions following 1%–60% of reviews. However, almost half the authorities (44%) reported reductions in between 1%–20% of cases, in whole or in part, meaning that at least 80% of packages remained, at least partially, double‐handed. Furthermore, more than 60% remained wholly double handed for 45% of authorities. Thus, our findings suggest that reductions may be at the lower end of the range reported in previous literature. One possible explanation is that as these reviews have been rolled out more widely and become more commonplace the number of packages suitable for reduction has decreased. That is, prior to this survey, those with the greatest potential for reductions were identified historically and have previously been reduced. This finding is limited as there was considerable variation between authorities with six authorities reporting that greater than 60% of packages were reduced in whole or in part; the reasons for these variations are not clear.

To our knowledge, this is the first survey of this type which has collated information on double‐handed homecare reviews across the majority of local authorities with social care responsibilities in England. It is therefore the most comprehensive presentation of data on this common practice, and this is a principal strength of the research. We had a pre‐specified minimum response rate of over fifty percent, and we were able to achieve this. The data from the pre‐coded questions should enable local authorities to compare their practices with others, forming something of a benchmark of practice in this area. However, it is possible that the authorities that responded were those that were most interested in this practice and therefore we are unable to reasonably infer in relation to practices in the non‐responding authorities. Furthermore, sending out the survey was delayed by the COVID‐19 pandemic and the first UK national lockdown. We were advised by our working group to delay sending this and even when we did, it is possible that this affected our response rate. Indeed, one authority contacted us stating they were unable to complete the survey due to the pressures of the pandemic. It is also possible that our survey did not reach the most appropriate person or team, for example, some authorities may also carry out such work as part of a reablement team or service. There are also some differences with terminology across local authorities in terms of what constitutes a ‘review’.

Our findings suggest that double‐handed homecare reviews are a prevalent area of practice within local authorities, which is set to increase. Despite this, robust evidence on outcomes for people receiving these reviews is limited and largely anecdotal. All authorities responding to the survey reported that reviews were completed in order to increase independence for the person using the service; however, we have been unable to find any research which has investigated the effect of these reviews on independence. Further research should explore this and might also compare outcomes for people whose care has been reduced with those whose care has not. Furthermore, the tensions which were alluded to with private homecare providers warrant further exploration. Further research should explore all stakeholders' views ‐ including homecare agencies and workers – and especially the views of people using the service who are still underrepresented in the literature.

## 5 CONCLUSION

This is the first study to collate data on local authority single‐handed care initiatives across local authorities in England. The findings have confirmed anecdotal evidence that they are a common practice within local authorities, which many local authorities plan to increase. The findings have highlighted the diversity in practices across local authorities alongside some key similarities. Given the number of reviews that are completed by local authorities, and the time spent completing them, further research should investigate the short, medium and long‐term outcomes for the person receiving the review.

## AUTHOR CONTRIBUTION

PW is the principal investigator for the study. JA‐T, CB, MG, CS, AR, TR, SW are co‐investigators on the research grant. LR is Senior Research Associate on the study. All authors contributed to the inception and design of the study and the interpretation of the data. PW led the analysis of the pre‐coded questions, and LR led the analysis of the free text questions. PW and LR drafted the manuscript. All authors commented on the manuscript.

## FUNDING INFORMATION

This report is independent research by the National Institute for Health Research (Research for Patient Benefit, Co‐production of best practice recommendations for local authority reviews of double‐handed homecare packages, NIHR200040). The views expressed in this publication are those of the author(s) and not necessarily those of the NHS, the National Institute for Health Research or the Department of Health and Social Care.

## Data Availability

The data that support the findings of this study are available from the corresponding author upon reasonable request.

## Endnotes

^1^
Note this was a multipoint answer where respondents could select more than one option.

^2^
Note this was a multipoint answer and respondents could select more than one option. 100% would represent that all those responders to this question choose that option.

^3^
Note this was a multipoint answer where respondents could select more than one option.
